# A hybrid hierarchical health monitoring solution for autonomous detection, localization and quantification of damage in composite wind turbine blades for tinyML applications

**DOI:** 10.1038/s41598-025-95364-5

**Published:** 2025-04-11

**Authors:** Nikhil Holsamudrkar, Shirsendu Sikdar, Akshay Prakash Kalgutkar, Sauvik Banerjee, Rakesh Mishra

**Affiliations:** 1 Department of Civil Engineering, Indian Institute of Technology, Bombay, Mumbai, 400076 India; 2 School of Computing and Engineering, University of Huddersfield, Queensgate, Huddersfield HD1 3DH UK

**Keywords:** Acoustic emission, Composites, Damage localization, Deep learning, Structural health monitoring, Wind turbine blades, TinyML application, Engineering, Mechanical engineering

## Abstract

Composites are widely used in wind turbine blades due to their excellent strength-to-weight ratio and operational flexibilities. However, wind turbines often operate in harsh environmental conditions that can lead to various types of damage, including abrasion, corrosion, fractures, cracks, and delamination. Early detection through structural health monitoring (SHM) is essential for maintaining the efficient and reliable operation of wind turbines, minimizing downtime and maintenance costs, and optimizing energy output. Further, Damage detection and localization are challenging in curved composites due to their anisotropic nature, edge reflections, and generation of higher harmonics. Previous work has focused on damage localization using deep-learning approaches. However, these models are computationally expensive, and multiple models need to be trained independently for various tasks such as damage classification, localization, and sizing identification. Also, the data generated due to AE waveforms at a minimum sampling rate of 1MSPS is huge, requiring tinyML enabled hardware for real time ML models which can reduce the size of cloud storage required. TinyML hardware can run ML models efficiently with low power consumption. This paper presents a Hybrid Hierarchical Machine-Learning Model (HHMLM) that leverages acoustic emission (AE) data to identify, classify, and locate different types of damage using the single unified model. The AE data is collected using a single sensor, with damage simulated by artificial AE sources (Pencil lead break) and low-velocity impacts. Additionally, simulated abrasion on the blade’s leading edge resembles environmental wear. This HHMLM model achieved 96.4% overall accuracy with less computation time than 83.8% for separate conventional Convolutional Neural Network (CNN) models. The developed SHM solution provides a more effective and practical solution for in-service monitoring of wind turbine blades, particularly in wind farm settings, with the potential for future wireless sensors with tiny ML applications.

## Introduction

As the world increasingly transitions to renewable energy sources, achieving net-zero emissions has moved from a theoretical goal to an actionable target embraced by numerous countries under the Paris Agreement^1,2^. Wind energy plays a significant role in this transition, contributing over 17% of global energy production—a figure expected to rise significantly in the coming decade^3^. The primary material used in wind turbine blade manufacturing is composites due to its favorable balance of cost, durability, and strength-to-weight ratio^4^. However, wind turbines often operate in harsh environmental conditions that can lead to various types of damage, including abrasion, corrosion, fractures, cracks, and delamination. Consequently, non-destructive testing (NDT) methods are crucial for ensuring the sustainability and reliability of wind turbine blades^5^.

Visual inspection is a commonly used method to superficially asses composite structures. For instance, Denhof et al.^6^ and He et al.^7^ developed complex convolutional neural network (CNN) models based on surface image data to differentiate between damaged and undamaged wind turbine blades. The research suggested utilizing drones to capture images, which are subsequently processed by the algorithm to identify and locate damage. Although this solution is effective, it is constrained to evaluating the structure’s current visible condition, potentially overlooking internal or micro damage and causing delays in repairs. A similar study employs advanced machine learning models to detect damage on blade surfaces using image data^8^. In another study, Wu et al.^9^ implemented a 3D digital image correlation (DIC) for vision-based continuous monitoring of wind turbine blades. However, DIC is highly sensitive to lighting conditions, making its practicality in real-world applications uncertain^10^. Moreover, internal damages cannot be detected through visual inspections or DIC alone.

Structural health monitoring (SHM) offers a more proactive solution by integrating sensors directly into the structural elements for continuous monitoring. Electromagnetic impedance (EMI) is a global method for damage detection in structural health monitoring (SHM) that uses piezoelectric transducers (PZT). It measures changes in materials’ conductance and transmittance properties to identify structural anomalies^11,12^. Another popular SHM technique, ultrasonic-guided waves (GW), is particularly effective due to its ability to cover long distances and detect minor defects, making them ideal for identifying and characterizing hidden structural issues^13–16^. Yang et al.^17^ used two GW techniques—non-linear acoustics and a “pitch-catch” method—to study wave behavior in small-scale turbine blades, successfully detecting and localizing impact damage. In another study, Tiwari and Raišutis^18^ analyzed scattered waveforms to identify disbond-type defects in wind turbine blades. Further, Shoja et al.^19^ expanded on GW methods by developing an icing index for detecting ice formation on blades, while Raišutis et al.^20^ used phase velocity variations to identify and size defects with high accuracy. Later, Dilek A. et al.^21^ demonstrated the use of IR laser vibrometers for capturing dynamic features of small-scale wind turbines, while Pan et al.^22^ observed a decrease in natural frequency in damaged blades, validated through numerical simulations. Vibration-based health monitoring using accelerometers is another popular and practical method adopted by many researchers^23–25^. These studies highlight the effectiveness of vibration data in early damage detection.

Another commonly utilized technique in SHM is acoustic emission (AE), known for its ease of use, wide range of applications, and potential for real-world implementation^26–28^. Unlike GW, AE is a passive technique that captures signals in the ultrasonic domain (>20kHz) generated by energy release from material deformations or damage mechanisms, such as fractures, delaminations, and cracks. In a study by Beale et al.^29^, a microphone array with an audible frequency range was placed on the blade’s surface for continuous monitoring. The authors utilized advanced signal processing methods to filter out noise generated during operation. AE sensors effectively attenuate operational noises since they occur in the low-frequency range. Traditional AE methods focus on two main challenges: source classification and source localization. Source classification identifies the damage mode (e.g., shear, flexure) or type (e.g., delamination, debonding), meanwhile source localizarion identifies the damage source location. In the similar lines, Tang et al.^30^ used AE to monitor full-scale wind turbine blades under fatigue loading, using sensors to localize damage based on signal arrival times. Whereas, Blanch and Dutton^31^ demonstrated the feasibility of transmitting AE data from rotating blades via broadband radio without losing resolution, while Joose et al.^32^ developed an AE-based certification test for locating critical damage areas in wind turbine blades. High energy and amplitude were identified as key indicators for detecting significant damage.

In AE source localization, the time of difference arrival (TODA) method, introduced by Tobias A.^33^, is commonly used and is adaptable for anisotropic materials like Fiber-reinforced polymer (FRP) composites. Further, other researchers applied this method to damage localization in composites^34,35^. Later, Sikdar et al.^36^ develooped advanced algorithms, such as particle swarm optimization, for damage localization using AE data. For complex geometries, a combination of AE and acousto-ultrasonic methods is recommended^37^. Also, if multiple media are present, then for localization, a novel method is developed by Zhou et al.^38^ considering the effect of refraction. All these methods have limitations, such as the need for multiple sensor arrays and reliance on accurate wave velocity profiles in composites^39^.

To address challenges from conventional localization algorithms, some researchers suggest zonal-based machine learning (ML) approaches for damage localization^40–42^. Additionally, machine learning can be used to predict or identify parameters through either classification or regression techniques^43,44^. Some studies have proposed enhancing location accuracy by utilizing machine learning-based graph construction methods in wind turbine blades^45^. However, applying a graph construction strategy to full-scale blades poses significant challenges, and further research is essential in this area. Also, Studies by Osa-uwagboe et al. have shown that ML models, particularly regression and ensemble learning algorithms, can effectively predict damage behavior using AE features, achieving high accuracy in quasi-static indentation and environmental exposure scenarios. For instance, models based on total energy absorption achieved R-squared values of about 0.99, while classification models identified damage modes with accuracy scores in the range of 86.4% to 95.9%. Here, KNN performed best for larger surface areas, capturing complex nonlinear relationships in damage evolution^46,47^. Further, The use of hybrid ML models were proposed by Azad and Kim, that integrates CNN and convolutional autoencoders (CAE) with support vector machines (SVM) for the damage diagnosis of laminated composites^48^. The proposed hybrid models showed better performace compared to conventional models. Further, to mitigate data scarcity and class imbalance issues in SHM data, Azad et al.^49^ proposed multi-class generative adversarial network (MC-GAN) coupled with CNN. It was reported that the the proposed framework achieves a mean accuracy of 99.72%.

Although AE is a powerful technique to detect and locate damages during the service life of composite structures, it generates huge volume of data due to high sampling rate of the data acquisition (minimum 1 MSPS). This can be mitigated by deploying TinyML enabled hardware which can run ML models in the real time for damage detection, and localization. However, this necessitates development of computationally efficient ML models which can perform multiple tasks within a single unified model. Also, further in-depth studies are required on AE-based damage classification and localization in complex composite structures, such as wind turbine blades, particularly under operational noise or combined damage conditions. Most of the studies are concentrated on composite plates or shells. However, a handful of studies are available that have focused on detecting and localizing combined damages in composite wind turbine blades. This study addresses this gap by applying a hierarchical hybrid machine learning model (HHMLM) to a small-scale wind turbine blade subjected to combined damage scenarios, such as environmental (abrasion) and impact/fracture/delamination. The model aims to classify damage types (impact vs. PLB), localize damage, and quantify damage size (abrasion length). Additionally, the study introduces additive white Gaussian noise (AWGN) to simulate the effects of operational noise on AE data. This also enlarges the dataset, enhancing the model’s predictive ability and reducing the risk of overfitting. The ultimate goal is to facilitate the deployment of AE sensors on full-scale wind turbine blades for in-service monitoring, with future studies focusing on wireless data collection and real time data processing using tinyML enabled hardware.

### Laboratory experiments

**Fig. 1:**
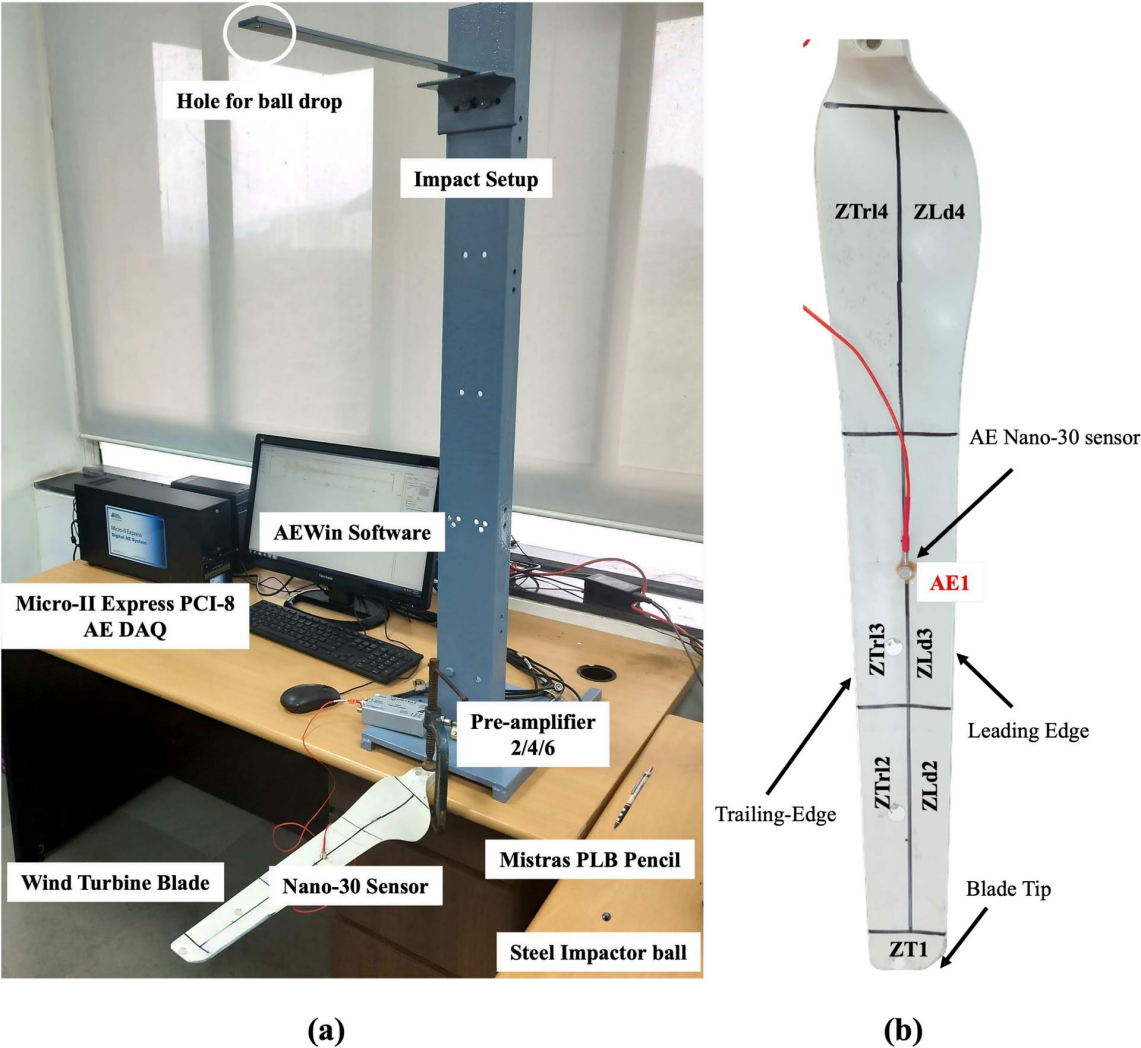
AE Experimental setup: (**a**) PLB and Impact tests, and (**b**) predefined damage/AE source zones.

**Fig. 2:**
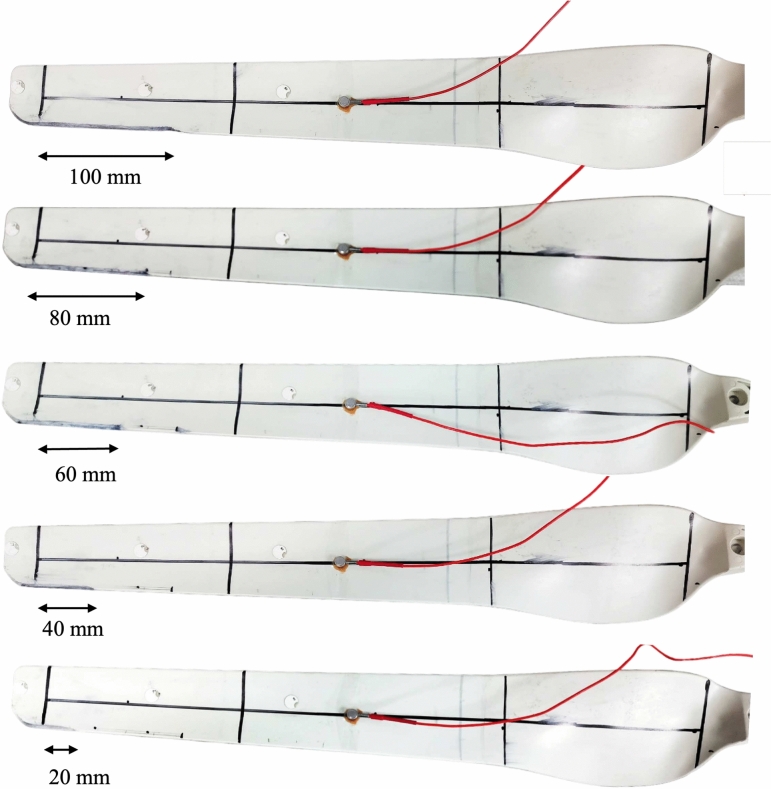
Abrasion damage of varying lengths created along the leading edge of the blade.

A laboratory experiment is conducted using a glass fiber reinforced polymer (GFRP) wind turbine blade sample, measuring 500 mm in length and scaled down to 1:20 of an industrial turbine blade. The blade is segmented into seven zones for damage localization, as depicted in Fig. 1. Two types of damage sources are considered: impact and fracture. The impact damage is simulated by dropping a steel ball impactor with a diameter of 6 mm, and a mass density of 7850 $kg/m^3$ from a height of 565 mm^40^. Fracture damage is generated using a Hsu-Nielsen pencil lead break (PLB) with 2H lead, which has a 0.5 mm diameter and a length of 3 mm. Figure 1 shows the complete experimental setup. Additionally, abrasion damage is induced at the blade’s leading edge, with lengths of 20 mm, 40 mm, 60 mm, 80 mm, and 100 mm, as illustrated in Fig. 2. The impact and PLB tests are conducted for each abrasion length. Abrasion is introduced by sanding the leading edge with 40-grit sandpaper to achieve an abrasion depth of 0.5 mm. Data is acquired using a MISTRAS Micro II Express PCI-8 acoustic emission system and a Nano-30 sensor placed at the blade’s center. Capturing damage across the entire full-scale turbine blade with a single sensor is challenging. Therefore, a separate study is ongoing to optimize multiple sensor placements, where each sensor will monitor a specific zone of the blade based on its attenuation characteristics. However, overall data capturing, filtering, and charachterization methodology developed in the present study remains the same even for more number of sensors. Further, the signals are pre-amplified using a 2/4/6 MISTRAS pre-amplifier with 40 dB amplification and acquired at a rate of 2 MSPS, following the Nyquist sampling theorem^50^. The peak definition time (PDT), hit definition time (HDT), and hit lockout time (HLT) are set at 60 $\mu$s, 120 $\mu$s, and 300 $\mu$s, respectively, based on signal characteristics observed on the oscilloscope. These values align with the past literature on composites^51^. The total time for each signal considered is 1000 $\mu$s.

### Signal processing and data preparation

**Fig. 3:**
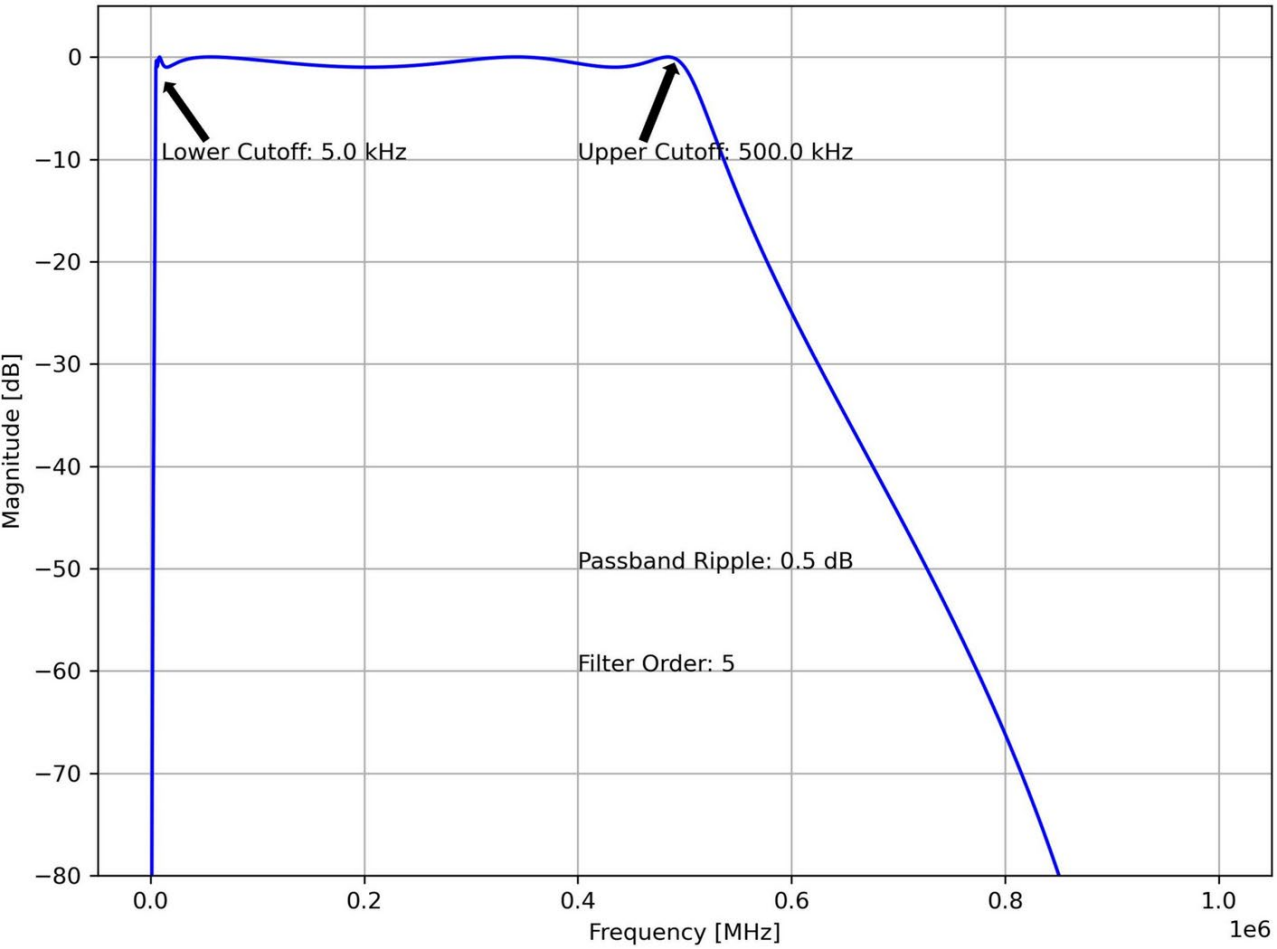
Designed Chebyshev Type I band-pass filter.

The pencil lead break is conducted with the utmost care, keeping the lead length, angle of lead with respect to specimen, and force of application constant. Despite this, signals with varying amplitudes were observed in the dataset, and noisy signals were generated due to double lead break, shear/frictional contact, etc. Also, in case of impact, rolling ball signals were also observed. A three-step filtering strategy is developed to mitigate unwanted noise generated during experiments or field implementation. A similar multi-step denoising strategy was suggested by Azad and Kim^52^. First, to avoid unnecessary data collection, a Chebyshev Type I band-pass filter is applied to eliminate signals not lying in the range of 5 kHz-500 kHz. The filter details are given in the Fig. 3.

Further, unwanted signals generated by friction and shear are filtered out using an amplitude-duration filter, as described in Table 1. The filter parameters are determined based on typical signal characteristics observed during experiments conducted in the lab. Various materials like sandpaper and hand contact are rubbed against the blade’s surface in these tests. Also, a table fan was used to mimic wind flow over the blade. The captured signals’ properties were analyzed, and filters were designed accordingly. It was observed that events such as shear, friction, or ball-rolling produced signals with low amplitude and long duration, which the filter could remove successfully.

**Table 1:** Amplitude-Duration (DA) Filter.

Condition	Amplitude (dB)	Duration ($\mu$s)
1	40 – 43	> 300
2	44 – 45	> 400
3	46 – 47	> 440
4	48 – 49	> 500
5	50 – 53	> 530
6	54 – 56	> 600
7	57 – 65	> 620

The final step is denoising signals using Singular Value Decomposition (SVD), Empirical Mode Decomposition (EMD), and Hilbert Spectral Analysis (HSA), which are complementary methods used for signal filtering and analysis. Each technique serves a distinct purpose, ultimately working together to clean, decompose, and analyze complex AE waveforms. First, SVD is applied to reduce noise in the signal by first reshaping the 1D AE signal into a Hankel matrix $X_H$. The SVD of $X_H$ is given by:

1 $$\begin{aligned} X_H = U \Sigma V^T \end{aligned}$$

where $U$ and $V$ are orthogonal matrices, and $\Sigma$ contains the singular values $\sigma _i$. The singular values, ordered as $\sigma _1 \ge \sigma _2 \ge \cdots$, represent the significance of each component. Smaller singular values often correspond to noise, while larger ones represent the signal features. By keeping only the largest $k$ singular values in a rank-$k$ approximation:

2 $$\begin{aligned} X_H \approx \tilde{X_H} = {\tilde{U}} {\tilde{\Sigma }} {\tilde{V}}^T \end{aligned}$$

we can reconstruct the signal while removing noise, retaining 90% of the original energy. This denoising process helps ensure that subsequent analysis on the signal is more accurate and less affected by noise. After the SVD-based denoising, Empirical Mode Decomposition (EMD) is applied to further decompose the signal into simpler components known as Intrinsic Mode Functions (IMFs). The signal $x(t)$ is decomposed as:

3 $$\begin{aligned} x(t) = \sum _{k=1}^{N} IMF_k(t) + r(t) \end{aligned}$$

where each $IMF_k(t)$ is an oscillatory mode that captures different time-scale variations in the signal, and $r(t)$ is the residual. The shifting process ensures that each IMF satisfies two conditions: 1) The number of zero crossings and extrema are equal or differ at most by one. 2) The mean value of the upper and lower envelopes (defined by the local maxima and minima) is zero. EMD is particularly useful for nonlinear and non-stationary signals. By isolating different frequency components in the form of IMFs, this step provides a clean representation of different features in the signal. Once the signal is decomposed into IMFs, Hilbert Spectral Analysis (HSA) is applied to perform a time-frequency analysis. The Hilbert Transform (HT) is used to generate an analytic signal $z(t)$ from a real signal $x(t)$:

4 $$\begin{aligned} z(t) = x_r(t) + j HT[x_r(t)] = a(t) e^{j \theta (t)} \end{aligned}$$

Here, $a(t)$ is the instantaneous amplitude, and $\theta (t)$ is the instantaneous phase. From this, the instantaneous frequency $f(t)$ can be calculated as:

5 $$\begin{aligned} f(t) = \frac{1}{2 \pi } \frac{d\theta (t)}{dt} \end{aligned}$$

The Marginal Hilbert Spectrum $h(f)$, analogous to the Fourier power spectrum, measures the amplitude contribution from each frequency over the entire time span:

6 $$\begin{aligned} h(f) = \int _{-\infty }^{\infty } {\mathfrak {R}} \left( a(t) \exp \left( 2 \pi j \int f(t) dt \right) \right) dt \end{aligned}$$

This step provides a detailed time-frequency representation, allowing for precise analysis of the signal’s frequency characteristics and transient events. Together, these steps form an effective pipeline for filtering and analyzing noisy, complex signals like AE waveforms. A similar approach was adopted by Holsamudrkar et al.^26^. Further, min-max normalization of the signal is carried out, i.e., by normalizing the signal’s amplitude in the range of [-1, 1] as follows:

7 $$\begin{aligned} X' = 2*\frac{X - X_{\text {min}}}{X_{\text {max}} - X_{\text {min}}}-1 \end{aligned}$$

Where $X'$ is the Normalized signal amplitude, $X$ is the signal amplitude at a given instance, $X_{\text {min}}$ is the minimum signal amplitude, and $X_{\text {max}}$ is the maximum signal amplitude. A threshold of 40 dB was applied to filter out background noise, which was determined through preliminary trial experiments. For each of the 7 zones, 6 abrasion lengths, and 2 damage types, at least 6 signals were recorded.

### Data augmentation

Data augmentation techniques expand a limited dataset, enhancing the model’s ability to generalize, which in turn allows it to be applied more reliably in real-world situations. The collected waveform data was augmented using two methods: adding white Gaussian noise (AWGN) of 5%, 10%, and 15% and using a mixup technique^53^. Mixup Data Augmentation (DA), introduced by Zhang et al.^54^, is utilized to create new training samples by linearly combining existing ones. The augmented signal, $s_{\text {mix}}$, is generated as follows:

8 $$\begin{aligned} s_{\text {mix}} = \lambda x_i + (1 - \lambda ) x_j \end{aligned}$$

In this expression, $x_i$ and $x_j$ are the original source signals from the same class, and $\lambda$ is a value randomly selected between 0 and 1, drawn from either a uniform or beta distribution. This strategy results in a new sample incorporating features from both original signals, adding more diversity to the training data, simulating real life scenarios. Using these two methods, a total dataset of 2520 signals is generated. This dataset was then divided into training, testing, and validation sets in an 80:10:10 ratio, with the testing and validation sets containing an equal representation of each class.

### Feature extraction HHMLM

The 1-dimensional signal represents the time and amplitude domain but misses out on frequency content. Meanwhile, the Fourier transform (FFT) represents the frequency and amplitude domain but doesn’t provide any inference on time-dependent data. The best way to represent all three domains is by using a continuous wavelet transform (CWT). Therefore, the CWT of the 1-dimensional signal is carried out, and scalogram images are extracted from it. Scalogram is an image-based representation of CWT, wherein the x-axis represents time features, the y-axis represents frequency features, and amplitude is represented by RGB color variation. The scales for the scalogram are converted to frequency features (pseudo-frequency) by using the relation pseudo-frequency = 1/scale. The Continuous Wavelet Transform (CWT) of a signal $x(t)$ is given by:

9 $$\begin{aligned} \text {CWT}(a, b) = \frac{1}{\sqrt{|a|}} \int _{-\infty }^{\infty } x(t) \, \psi ^*\left( \frac{t - b}{a}\right) \, dt \end{aligned}$$

Where $x(t)$ is the signal as a function of time $t$, $a$ is the scale parameter, $b$ is the translation parameter, $\psi ^*(t)$ is the complex conjugate of the mother wavelet, and $\frac{1}{\sqrt{|a|}}$ is the normalization factor. The present study uses a Morlet wavelet as a mother wavelet, and its complex conjugate is given by:

10 $$\begin{aligned} \psi ^*(t) = \frac{1}{\pi ^{1/4}} e^{-i \omega _0 t} e^{-\frac{t^2}{2}} \end{aligned}$$

Where $t$ is the time variable, $\omega _0$ is the central frequency of the wavelet, $e^{-i \omega _0 t}$ is the complex conjugate of the sinusoidal function, $e^{-\frac{t^2}{2}}$ is the Gaussian envelope, and $\frac{1}{\pi ^{1/4}}$ is the normalization factor.

**Fig. 4:**
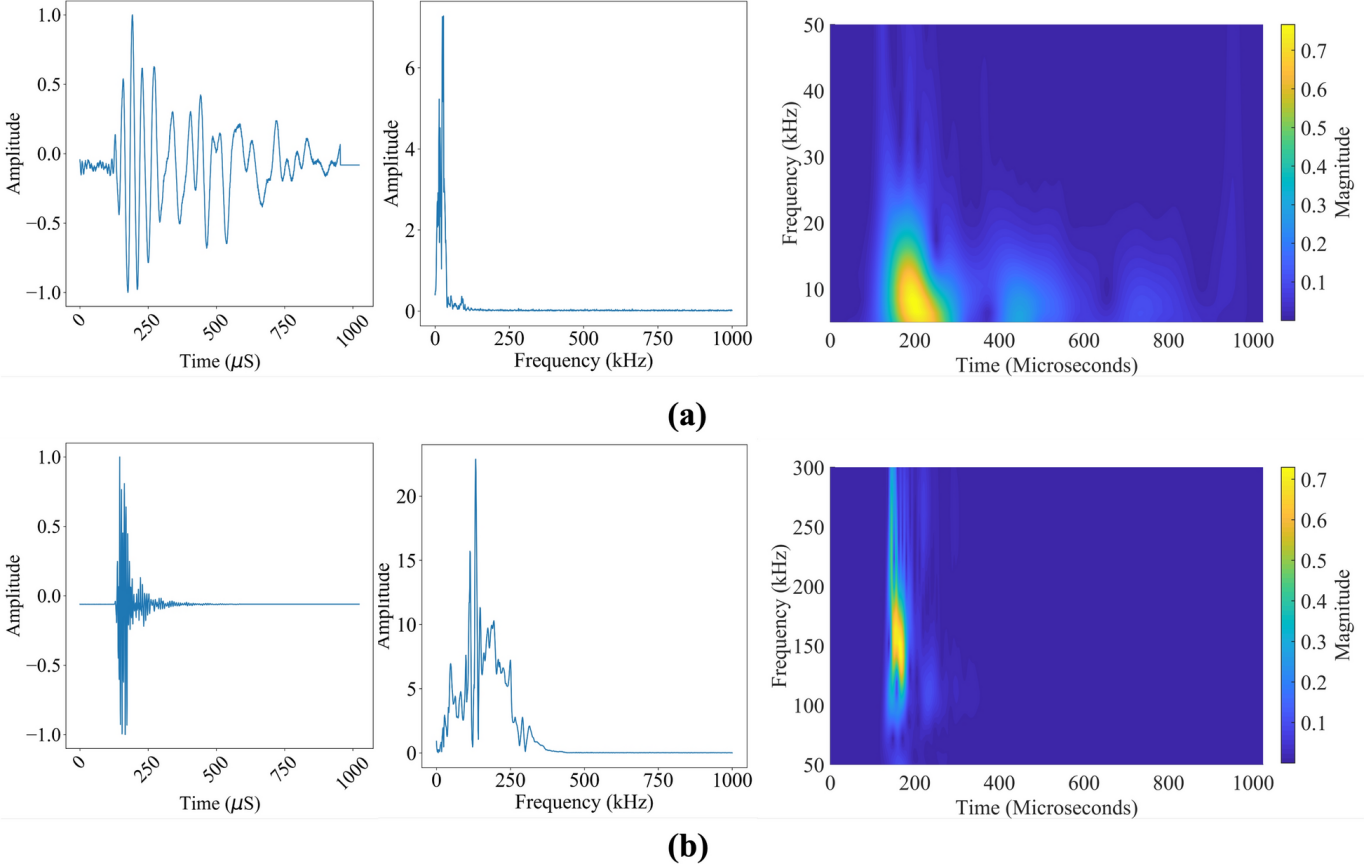
Original signal, its FFT, and CWT for (a) Impact, and (b) PLB damage.

The representative signals for impact and PLB, along with their FFT and CWT, are shown in Fig. 4. Since CNN models typically perform better when images have a 1:1 aspect ratio, all image data were extracted with this fixed aspect ratio, omitting axis ticks, labels, and color bars. Additionally, the axis limits were standardized, with the y-axis set from 0 to 500 kHz and the x-axis from 0 to 1000 $\mu$s. The extracted data were organized into folders following a hierarchical structure: starting with damage type, then damage location, and finally, abrasion length.

### Designed HHMLM architecture

**Figure 5:**
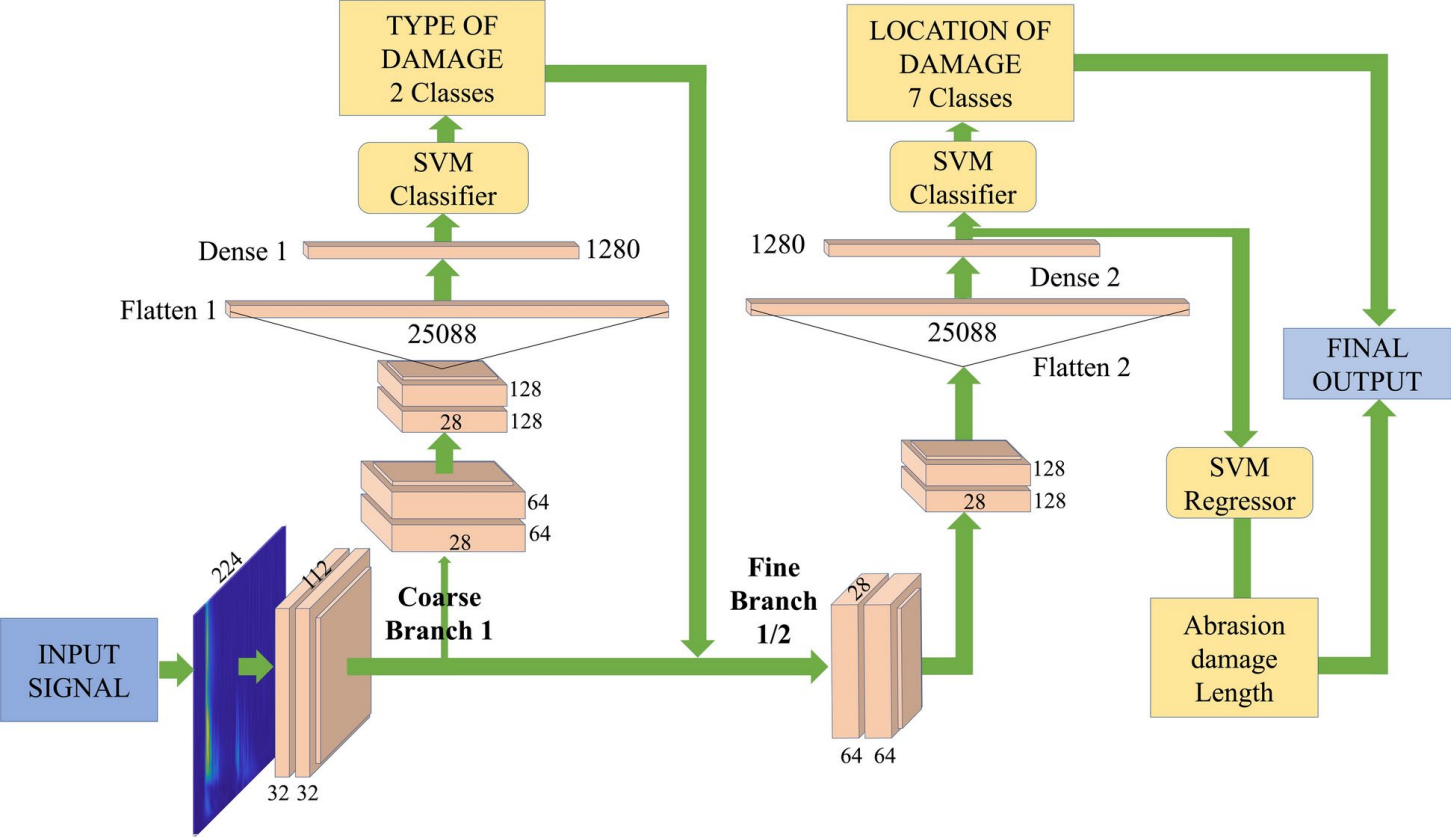
Designed HHMLM architecture.

The developed HHMLM architecture leverages the advantages of both CNN and SVM for classification and regression tasks. This strategy is particularly useful for handling complex hierarchical data structures, where extracting local features and global classification are essential. The architecture operates in two phases: first, the CNN extracts features, and then the SVM performs classification or regression based on those features. One key benefit of this hybrid CNN-SVM model over traditional deep CNNs is its ability to reduce the number of required convolutional layers while significantly enhancing classification and regression performance. The reduction in convolution layers reduces the overall computation requirement thereby optimizing tinyML hardware processing. The structure of the HHMLM model is illustrated in Fig. 5. The model functions in a hierarchical manner, first identifying the damage type using the coarse branch. Based on the output from the coarse branch, the damage location and abrasion length are determined through fine branch 1 or fine branch 2. The abrasion length is predicted using an SVM regressor, while the location is identified using an SVM classifier. The performance of this HHMLM model is then compared with the traditional VGG16 CNN model, as proposed by Simonyan et al.^55^, by training each task independently without any hierarchy structure. The results from both models are presented in section 7.

### Mathematical framework of HHMLM

#### CNN part

Let $X$ be the input data (RGB image in the present model). The CNN transforms the input $X$ into a feature vector $F$ through a series of convolution layers:

11 $$\begin{aligned} F = f_{\text {CNN}}(X; \theta ) \end{aligned}$$

The convolutional layer is defined as:

12 $$\begin{aligned} z_{i,j,k} = \sum _{m=1}^{M} \sum _{n=1}^{N} \sum _{p=1}^{P} w_{m,n,c,k} \cdot x_{i+m-1, j+n-1, c} + b_k \end{aligned}$$

Where $z_{i,j,k}$ is the Output feature map at position $(i,j)$ in the $k^{th}$ channel, $w_{m,n,c,k}$ is the weight of the filter at position $(m,n)$ in the $c^{th}$ input channel, and $k^{th}$ output channel, $x_{i+m-1, j+n-1, c}$ is the input feature map at position $(i+m-1, j+n-1)$ in the $c$-th channel, $b_k$ is the bias term for the $k^{th}$ output channel, $M \times N$ is size of the convolutional filter, and $P$ is the number of input channels. In this study, CNN uses three input channels (RGB) and features three convolutional layers with sizes of 32, 64, and 128 filters. Convolution involves sliding a small window, or filter, over an image to detect patterns. Activation functions are applied after each layer to add non-linearity to the convolutional layers, which is crucial for tackling complex, real-world tasks. The activation function used is Leaky ReLU, defined as:

13 $$\begin{aligned} a_{i,j,k} = \text {Leaky ReLU}(z_{i,j,k}) = {\left\{ \begin{array}{ll} z_{i,j,k} & \text {if } z_{i,j,k} \ge 0 \\ \alpha z_{i,j,k} & \text {if } z_{i,j,k} < 0 \end{array}\right. } \end{aligned}$$

$a_{i,j,k}$ is the output after applying the Leaky ReLU activation function, $z_{i,j,k}$ is the input value to the activation function (from the convolutional layer), $\alpha$ is the slope for the negative part of the function, typically a small positive value. For the current model, $\alpha = 0.01$ is considered. Leaky ReLU helps avoid discarding potentially important information by providing a non-zero output for negative input values, which can be beneficial in noisy or outlier-prone data scenarios, often outperforming ReLU. Pooling in CNNs is essential for improving computational efficiency, decreasing the dimensionality of feature maps, and boosting the network’s ability to generalize by concentrating on key features and adding some translation invariance. Additionally, the max pooling layer is defined by:

14 $$\begin{aligned} p_{i,j,k} = \max _{m,n} \left( a_{i+m-1, j+n-1, k} \right) \end{aligned}$$

$p_{i,j,k}$ is the output of the pooling layer at position $(i,j)$ in the $k$-th channel, $a_{i+m-1, j+n-1, k}$ is the input feature map at position $(i+m-1, j+n-1)$ in the $k$-th channel, $m,n$ are the indices over the pooling window size. A pooling size of 2 x 2 is considered with a stride of 2. To keep the output size the same as the input, padding of type ’same’ is applied. After three convolution layers, features are converted into 1D vectors, known as the flattening operation, given by:

15 $$\begin{aligned} D = \text {Flatten}(p) \end{aligned}$$

Where $D$ is the Flattened 1D feature vector, $p$ is the input feature map from the last pooling layer. Further, the dense layer is introduced, which is fully connected to the flattened layer and is computed as:

16 $$\begin{aligned} F_j = \sigma \left( \sum _{i=1}^{N} w_{i,j} \cdot D_i + b_j \right) \end{aligned}$$

Where $F_j$ is the output of the dense layer at the $j$-th neuron, $w_{i,j}$ is the weight between the $i$-th input feature and the $j$-th neuron, $D_i$ is $i$-th input feature from the flattened layer, $b_j$ is the bias term for the $j$-th neuron, $\sigma (x)$ is the activation function which is leaky ReLu in the present case.

A CNN’s first convolutional layer usually learns to detect simple edges (e.g., horizontal, vertical, diagonal) in different orientations. These edges are the fundamental building blocks of an image, allowing the network to differentiate between light and dark regions. As we progress to the second and third convolutional layers, the network combines these edges to recognize more complex textures and simple patterns, such as corners, curves, and basic shapes. These layers still focus on relatively small and local details. The network also begins to pick up on color gradients and simple textures, which help distinguish different image regions. In the HHMLM model, the CNN acts as a powerful feature extractor, thereby learning hierarchical representations of the input data, while the SVM is utilized for classification or regression based on these features.

#### SVM for classification

The One-vs-One (OvO) strategy for multiclass classification is used because it handles complex decision boundaries between classes. Each classifier in OvO deals with a balanced binary classification problem, which can improve accuracy and robustness. Additionally, OvO can mitigate issues related to class imbalance that might affect One-vs-All (OvA) classifiers. In OvO SVM classification, separate binary classifiers are trained for each pair of classes. Consider, for a dataset with $K$ classes, $$\left( {\begin{array}{c}K\\ 2\end{array}}\right)$$ binary SVM classifiers are trained. The prediction for a new input $X$ is based on the majority vote from all binary classifiers. For a binary classifier trained between class $c$ and class $d$, the prediction function is given by:

17 $$\begin{aligned} y_{c,d}(X) = \text {sign}\left( \sum _{i=1}^{n} \alpha _i^{c,d} y_i^{c,d} K(f_{\text {CNN}}(X; \theta ), f_{\text {CNN}}(X_i; \theta )) + b^{c,d} \right) \end{aligned}$$

where $y_{c,d}(X)$ is the predicted class label ($+1$ for class $c$ and $-1$ for class $d$), $\alpha _i^{c,d}$ are the Lagrange multipliers obtained from the SVM optimization problem for the classifier distinguishing between class $c$ and class $d$, $y_i^{c,d}$ is the true label for the $i$-th training sample, taking values $+1$ or $-1$ depending on whether the sample belongs to class $c$ or class $d$, $K(f_{\text {CNN}}(X; \theta ), f_{\text {CNN}}(X_i; \theta ))$ is the kernel function measuring the similarity between the CNN-extracted features of input $X$ and the $i$-th training sample $X_i$, and $b^{c,d}$ is the bias term specific to the binary classifier between class $c$ and class $d$. The final prediction is made by applying all binary classifiers and selecting the class that receives the most votes:

18 $$\begin{aligned} y(X) = \arg \max _{c} \sum _{d \ne c} {\textbf{1}}\{y_{c,d}(X) = +1\} \end{aligned}$$

Where ${\textbf{1}}\{y_{c,d}(X) = +1\}$ is an indicator function that is 1 if the classifier $y_{c,d}(X)$ predicts class $c$, and 0 otherwise. For each binary classifier between class $c$ and class $d$, the dual optimization problem is defined as:

19 $$\begin{aligned} \min _{\alpha ^{c,d}} \left( \frac{1}{2} \sum _{i=1}^{n} \sum _{j=1}^{n} \alpha _i^{c,d} \alpha _j^{c,d} y_i^{c,d} y_j^{c,d} K(F_i, F_j) - \sum _{i=1}^{n} \alpha _i^{c,d} \right) \end{aligned}$$

Where $\alpha _i^{c,d}$ and $\alpha _j^{c,d}$ are the Lagrange multipliers for the binary classifier between classes $c$ and $d$, $y_i^{c,d}$ and $y_j^{c,d}$ are the true class labels for the $i$-th and $j$-th training samples, $K(F_i, F_j)$ is the kernel function applied to the CNN-extracted features $F_i$ and $F_j$, and the first term represents the model complexity, accounting for pairwise interactions between training samples, and the second term represents the error term. The constraints for the dual optimization problem are:

20 $$\begin{aligned} \sum _{i=1}^{n} \alpha _i^{c,d} y_i^{c,d} = 0, \quad 0 \le \alpha _i^{c,d} \le C \end{aligned}$$

Where $\sum _{i=1}^{n} \alpha _i^{c,d} y_i^{c,d} = 0$ ensures the balance of the Lagrange multipliers for the binary classifier, and $0 \le \alpha _i^{c,d} \le C$ constrains each Lagrange multiplier between 0 and $C$, with $C$ being the regularization parameter controlling the trade-off between maximizing the margin and minimizing classification error.

#### SVM for regression

For SVM regression, the prediction function is given by:

21 $$\begin{aligned} y(X) = \sum _{i=1}^{n} (\alpha _i - \alpha _i^*) K(f_{\text {CNN}}(X; \theta ), f_{\text {CNN}}(X_i; \theta )) + b \end{aligned}$$

Where $y(X)$ is the predicted continuous value for input $X$, $\alpha _i$ and $\alpha _i^*$ are the Lagrange multipliers associated with the training samples $X_i$, $K(f_{\text {CNN}}(X; \theta ), f_{\text {CNN}}(X_i; \theta ))$ is the kernel function that measures the similarity between the CNN-extracted features of input $X$ and the $i$-th training sample $X_i$, and $b$ is the bias term that adjusts the prediction. The dual optimization problem for SVM regression is defined as:

22 $$\begin{aligned} \min _{\alpha , \alpha ^*} \left( \frac{1}{2} \sum _{i=1}^{n} \sum _{j=1}^{n} (\alpha _i - \alpha _i^*)(\alpha _j - \alpha _j^*) K(F_i, F_j) - \sum _{i=1}^{n} (\alpha _i - \alpha _i^*) y_i \right) \end{aligned}$$

Where $\alpha _i$ and $\alpha _i^*$ are Lagrange multipliers that correspond to the deviations from the $\epsilon$-insensitive loss margin on both sides of the true value, $K(F_i, F_j)$ is the kernel function applied to the CNN-extracted features $F_i$ and $F_j$ from training samples $X_i$ and $X_j$, The first term represents the model’s complexity in terms of all pairwise interactions between training samples, and the second term represents the deviation from the true values, which the optimization aims to minimize. The constraints for the dual optimization problem in SVM regression are:

23 $$\begin{aligned} \sum _{i=1}^{n} (\alpha _i - \alpha _i^*) = 0, \quad 0 \le \alpha _i, \alpha _i^* \le C \end{aligned}$$

Where $\sum _{i=1}^{n} (\alpha _i - \alpha _i^*) = 0$ ensures the balance of the deviations from the true value in both directions, and $0 \le \alpha _i, \alpha _i^* \le C$ constrains the Lagrange multipliers $\alpha _i$ and $\alpha _i^*$ between 0 and $C$, where $C$ controls the trade-off between the flatness of the regression function and the tolerance to deviations larger than $\epsilon$. The current study employs the Adaptive Moment Estimation (ADAM) optimizer with a learning rate of 0.00005 to address optimization challenges. ADAM works on a gradient-based optimization algorithm that adjusts the model’s weights based on the error provided by the cross-entropy loss as given by MacKay^56^.

#### Hyperparameters and training methodology

The overall model’s hyperparameters include the initializer, optimizer, and activation functions. The CNN component includes learning rate, filter size, pooling size, and stride, whereas the SVM component’s hyperparameters include the regularization parameter *C*, gamma ($\gamma$) , and epsilon ($\epsilon$). These are optimized using a grid search method. Specific details of the hyperparameters used in the model are provided in Table 2. $\gamma$, used in the Radial Basis Function (RBF) kernel, controls the influence of individual training points on the decision boundary, with a higher $\gamma$ leading to a more complex, localized boundary, and a lower $\gamma$ resulting in a smoother, more generalized boundary. $\epsilon$ defines a margin of tolerance where errors within this range are not penalized, with smaller $\epsilon$ values making the model more sensitive to deviations, while larger values allow for greater error tolerance. Both parameters significantly affect the complexity and performance of the model.

**Table 2:** Hyperparameters for the HHMLM.

Overall	CNN	SVM
Initializer = He^57^	Learning Rate = 0.00005	*C* = 0.5
Optimizer = ADAM^58^	Filter Size = 3 x 3	$\gamma$ = 0.01
Activation = Leaky RELU	Stride = 2	$\epsilon$ = 0.2
	Pooling Size = 2 x 2	

In the HHMLM, the coarse branch’s output is the fine branches’ input for damage localization and size identification (abrasion length). The abrasion length is predicted with an SVM regressor, and the location is identified with an SVM classifier, both utilizing the same CNN feature extraction branch. Previous research has adopted a similar method where features from the coarse branch and conditional branches are used as inputs for the fine branch features^59^. Additionally, this approach involves using a loss function that is a weighted average of the losses from the fine and coarse branches. The present study employs a similar strategy for the fine branch. Consequently, the loss function for the fine branch is defined as follows:

24 $$\begin{aligned} Loss_{avg}=\lambda * Loss_{fine} + (1-\lambda ) * Loss_{coarse} \end{aligned}$$

**Figure 6:**
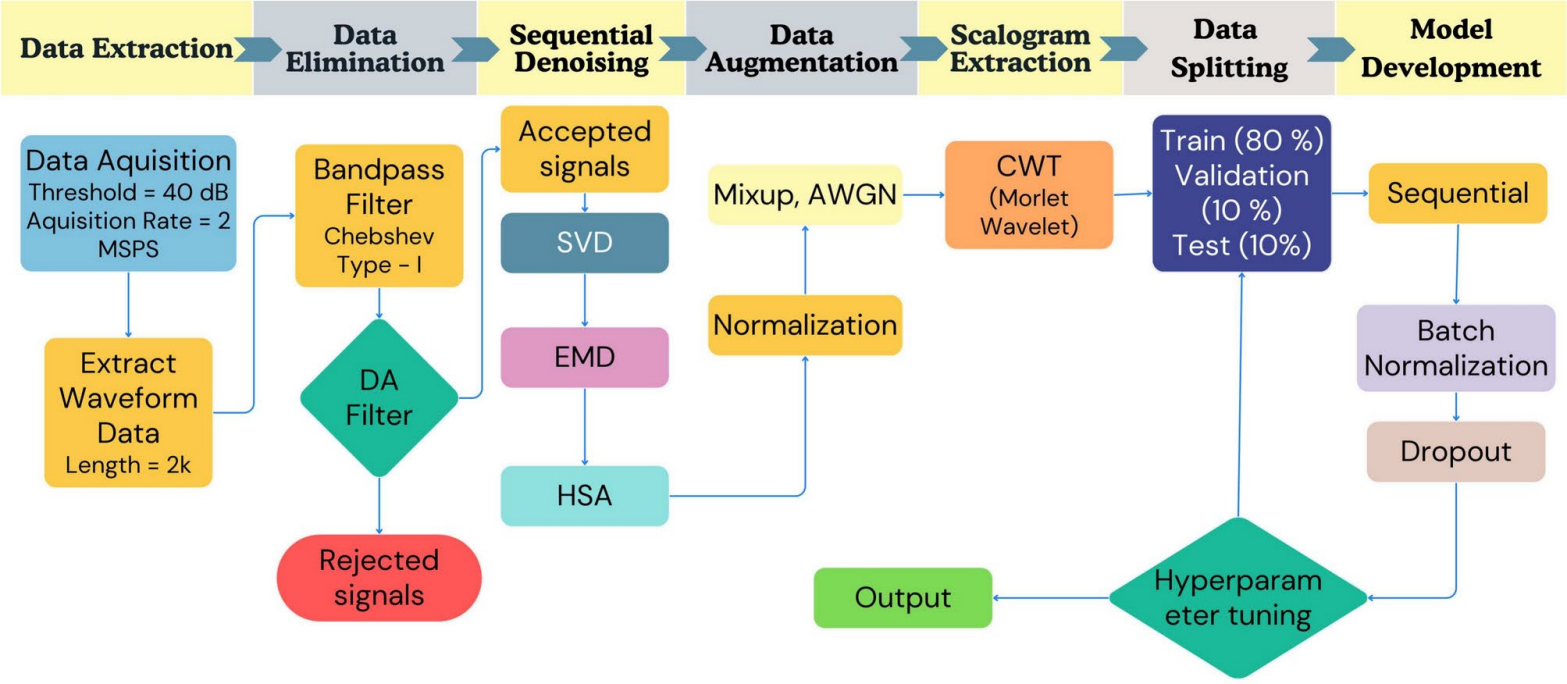
Overall Workflow for the Model.

Where $\lambda$ is taken as 0.4, which is optimized based on the performance of the fine branch prediction, the loss function used is cross-entropy, which is input to the ADAM optimizer for updating model weights. The overall workflow for the Model is illustrated in Fig. 6.

The HHMLM parameters include several key components for enhancing performance and robustness. Image-based features were extracted using continuous wavelet transform (CWT) to train the model. Data augmentation techniques such as Mixup and Gaussian noise addition were applied to expand the dataset. He et al. proposed initialization method^57^ which was employed to address vanishing and exploding gradient issues associated with the leaky ReLU activation function. Regularization techniques, including batch normalization^60^ and dropout^61^, were implemented to reduce overfitting and improve model performance. The ADAM optimizer^58^ was chosen to enhance convergence during training. Hyperparameters were optimized using a randomized grid search method, as detailed in Table 2. Additionally, an early stopping callback was utilized to halt the training process when validation metrics ceased to improve. A stratified shuffle cross-validation technique was used to mitigate the risk of sampling bias^62^. This method combines shuffle and stratified fold split, providing a more robust model evaluation. The model’s performance was assessed using the “loss” metric.

## Results and discussion

This section compares the results of the HHMLM to the conventional VGG16 model using the F1-score and Matthews correlation coefficient (MCC). Both models are developed using the Tensorflow library in Python. Additionally, model interpretability is evaluated using Shapely additive explanations (SHAP).

**Figure 7:**
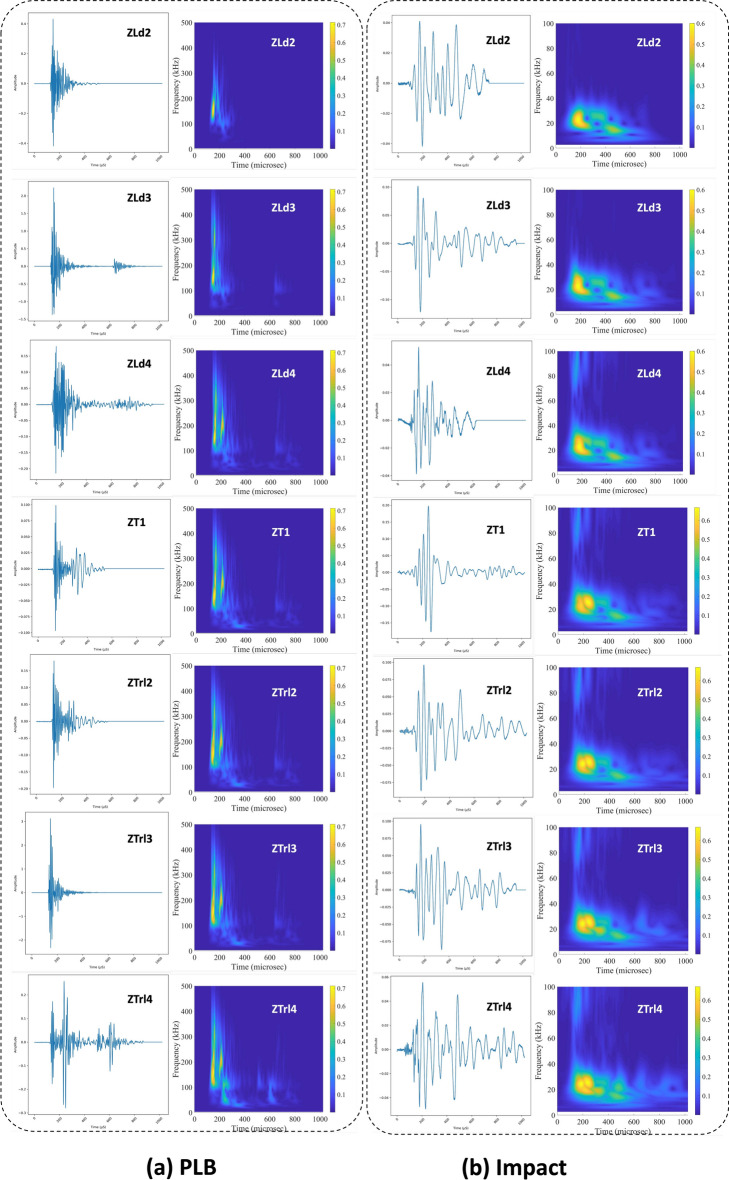
Signals and their continuous wavelet transforms (CWT) for different types of damage across various zones.

The extracted signals and preprocessing are crucial in classification and regression accuracy. It was observed that impact signals exhibit low-frequency features, while PLB signals display high-frequency characteristics. Likewise, for localization, each turbine blade zone has distinct signal features, as shown in Fig. 7, and these features vary for every abrasion length. This aids the model in identifying unique features specific to each class/zone.

### Accuracy and loss

**Fig. 8:**
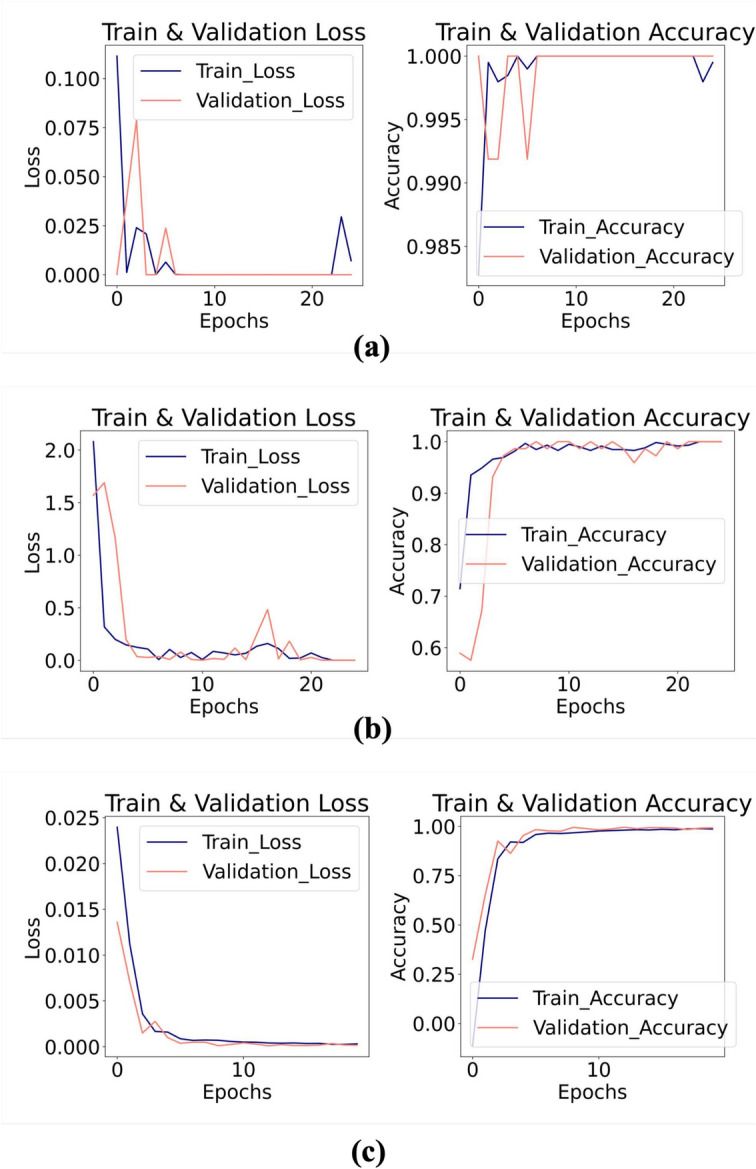
Training, validation loss, and accuracy curves for (**a**) coarse branch (damage type), (**b**) fine branch 2 (damage location), and (**c**) regression branch (abrasion length).

The accuracy and loss curves for the HHMLM, as shown in Fig. 8, demonstrate the model’s performance over 25 epochs. Training for the model was halted at 20 epochs for the classification task and 15 epochs for the regression task to prevent overfitting. During the training of the hybrid model, both training and validation losses initially dropped sharply before leveling off, indicating effective learning, while the corresponding accuracy curves showed a rapid increase, reflecting a strong generalization of the trained model. The metrics used for classification tasks (damage type and location) were the MCC and F1 score, while the $R^2$ score was employed as the performance metric for the regression task (abrasion length).

#### Matthews correlation coefficient (MCC)

The MCC, developed by Matthews^63^, is a correlation coefficient between the observed and predicted classifications; it returns a value between -1 and +1. A coefficient of +1 represents a perfect prediction, 0 is no better than a random prediction, and -1 indicates total disagreement between prediction and observation. For multiclass classification, the MCC is defined as:

25 $$\begin{aligned} \text {MCC} = \frac{TP \cdot TN - FP \cdot FN}{\sqrt{(TP + FP)(TP + FN)(TN + FP)(TN + FN)}} \end{aligned}$$

Where TP is the number of correctly predicted positive instances, TN is the number of correctly predicted negative instances, FP is the number of instances incorrectly predicted as positive, and FN is the number of instances incorrectly predicted as negative.

#### F1 score

The F1 Score^64^ is the harmonic mean of precision and recall. It reaches its best value at 1 and worst at 0. The F1 Score considers both the precision (the number of correct positive results divided by the number of all positive results) and the recall (the number of correct positive results divided by the number of positives that should have been retrieved). F1 score is defined as:

26 $$\begin{aligned} F1 = \frac{2 \cdot TP}{2 \cdot TP + FP + FN} \end{aligned}$$

**Fig. 9:**
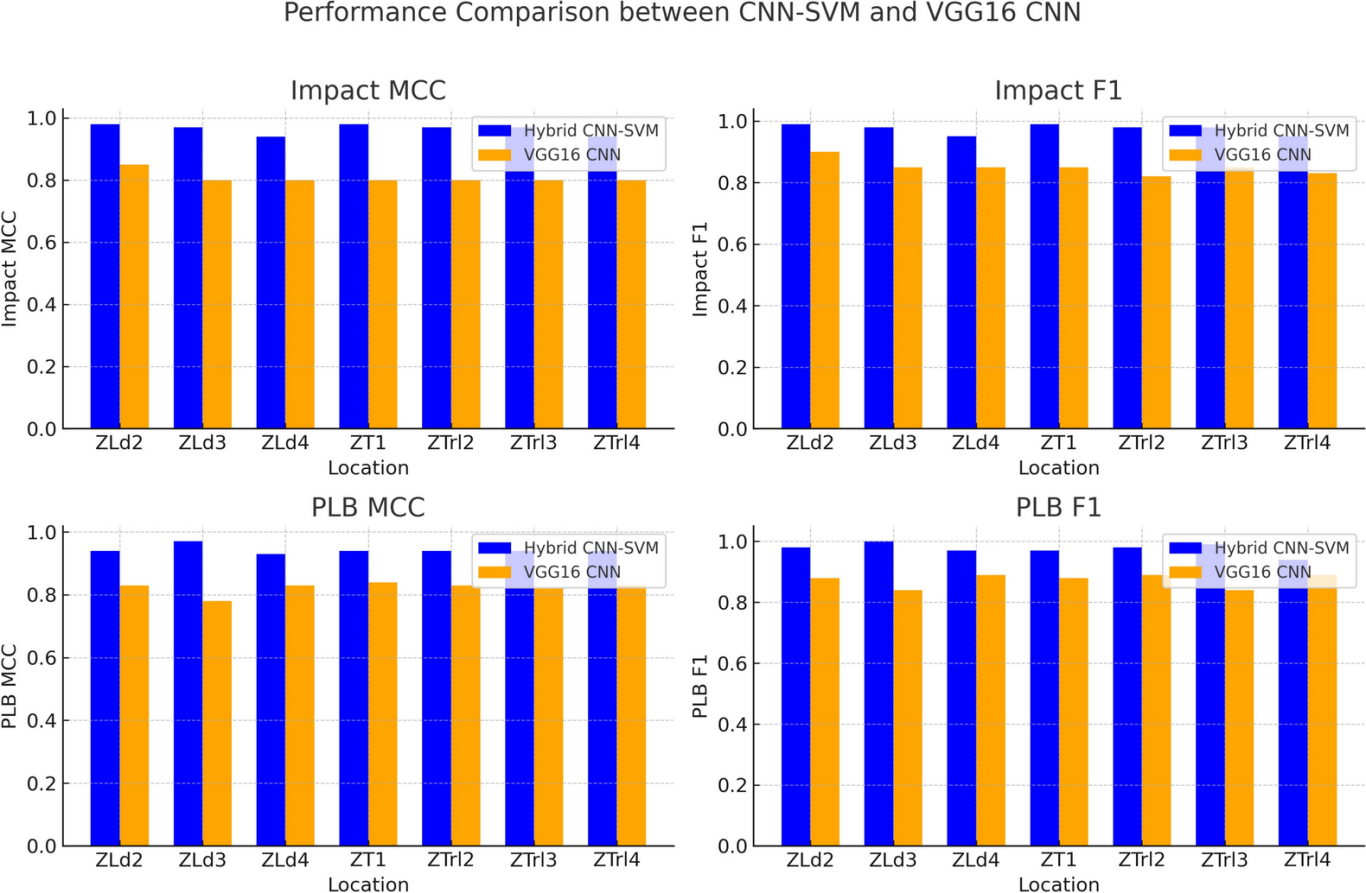
Performance comparison between HHMLM and VGG16.

**Fig. 10:**
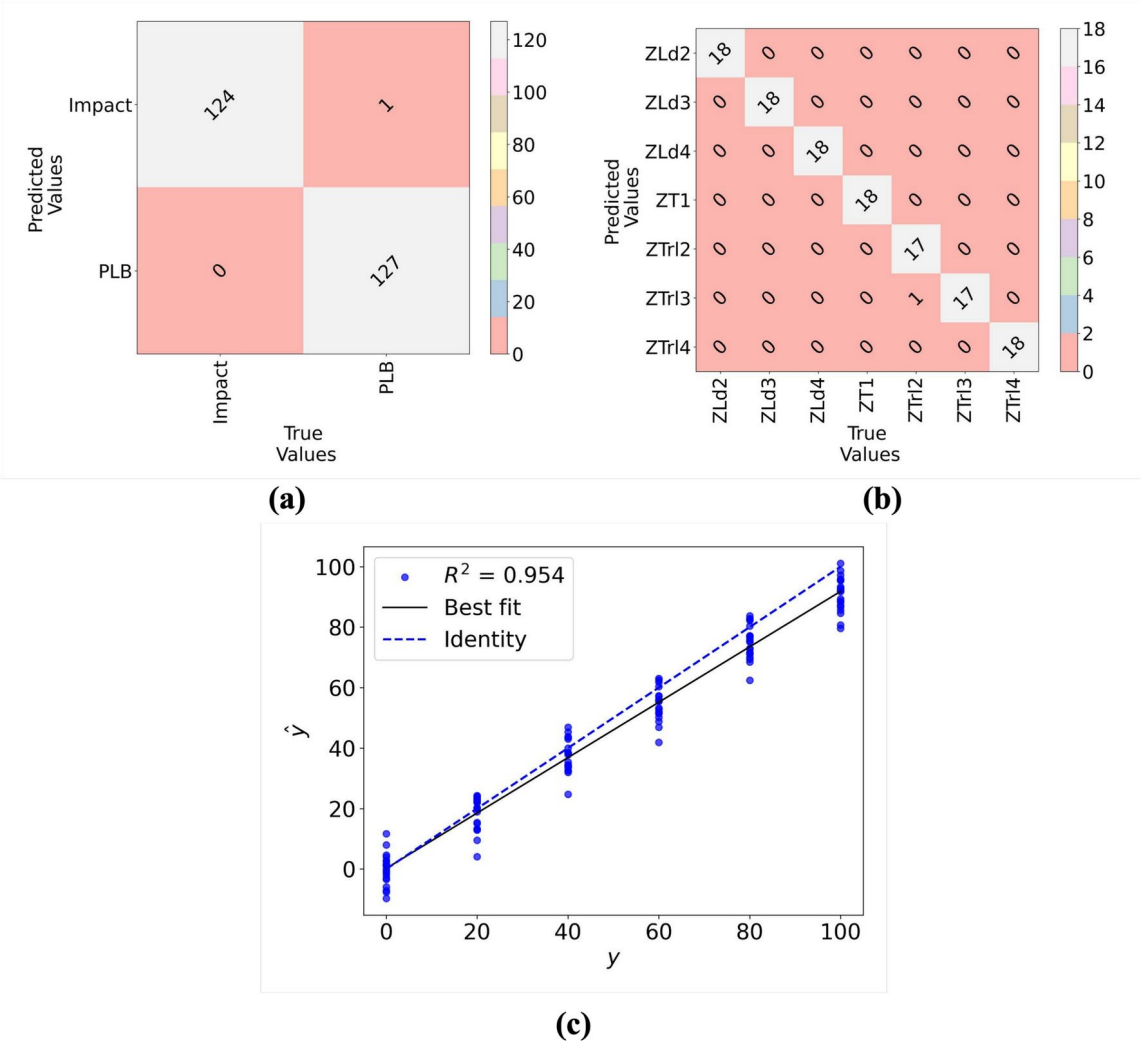
(**a**) Test Confusion matrix for coarse branch (damage type), (**b**) Test confusion matrix for fine branch 2 (damage location), and (**c**) prediction error for regression branch (abrasion length).

For damage type classification (Impact/PLB), the HHMLM achieved MCC and F1 scores of 0.99 and 1.0, respectively, compared to 0.89 and 0.90 for the VGG16 CNN model. The HHMLM showed higher accuracy in the coarse branch than in the fine branch. The detailed performance metrics for damage location are given in Fig. 9, which highlights the good performance of the HHMLM. For the regression task, the $R^2$ scores were 0.95 for the HHMLM and 0.82 for the conventional VGG16 regressor, indicating better performance of the fine branch than the independent VGG16 regressor. To better understand the HHMLM performance, the test confusion matrix, and prediction error were employed as represented in Fig. 10. It can be worth noting that the model overfitting was prevented using the callback method for early stopping in addition to the regularization technique. The overall performance of the HHMLM demonstrates an accuracy of 96.4%, while the VGG16 model achieves an accuracy of 83.8%.

### Model interpretability using SHAP

Shapley values, originating from game theory, are utilized to allocate payouts based on individual contributions to the total outcome. In the context of machine learning, Shapley values measure the global importance of features in a model’s prediction and are introduced by Lundberg et al.^65^. When applied to a HHMLM, Shapley values provide insights into how each feature extracted by the CNN contributes to the final classification made by the SVM. It is a widely used tool among researchers for interpreting machine learning models^66,67^. Given a cooperative game with $d$ players (features), the Shapley value for feature $j$ is defined as:

**Fig. 11:**
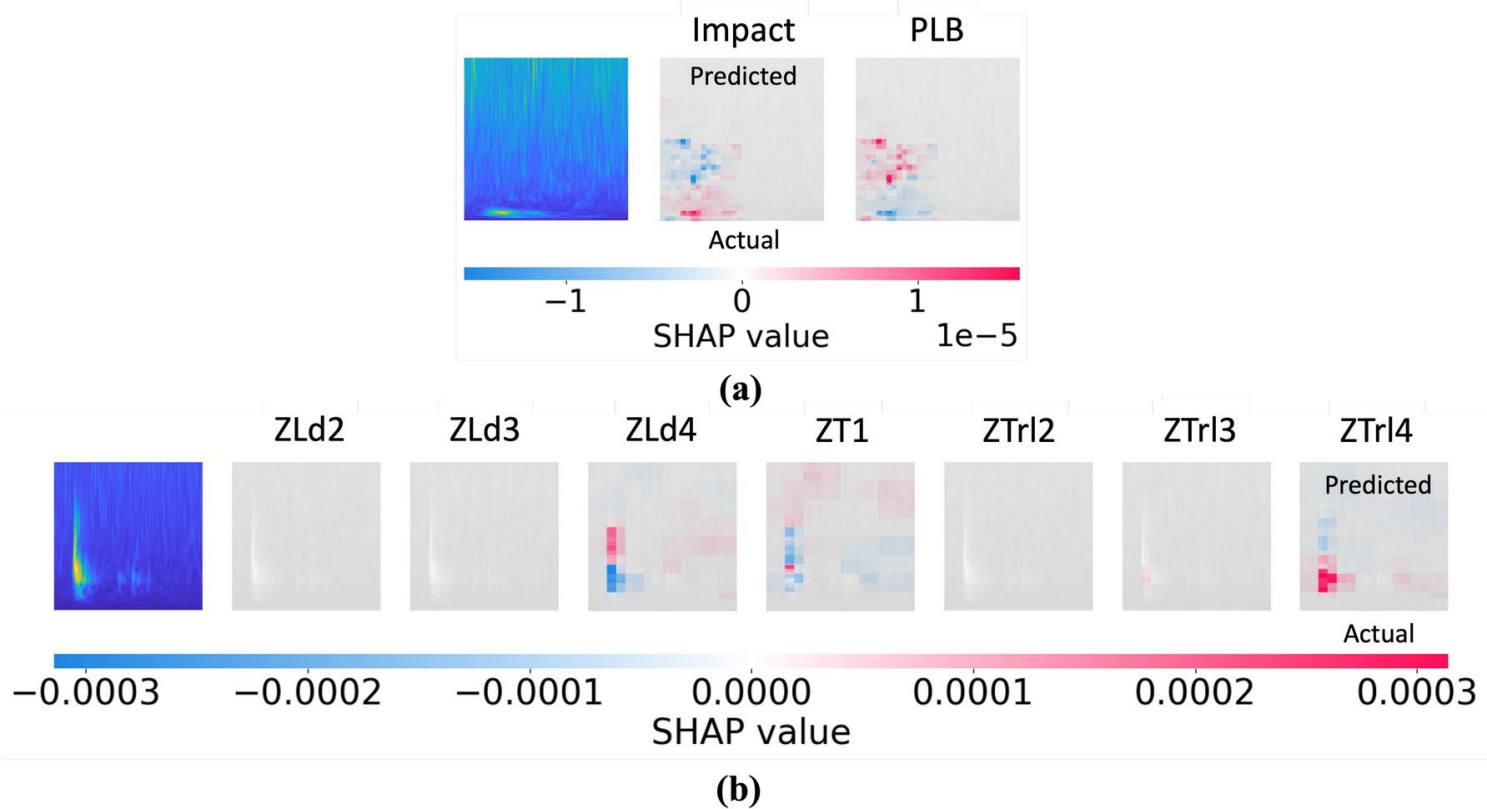
SHapley plots for (a) coarse branch (damage type), and (b) fine branch 2 (damage location).

**Fig. 12:**
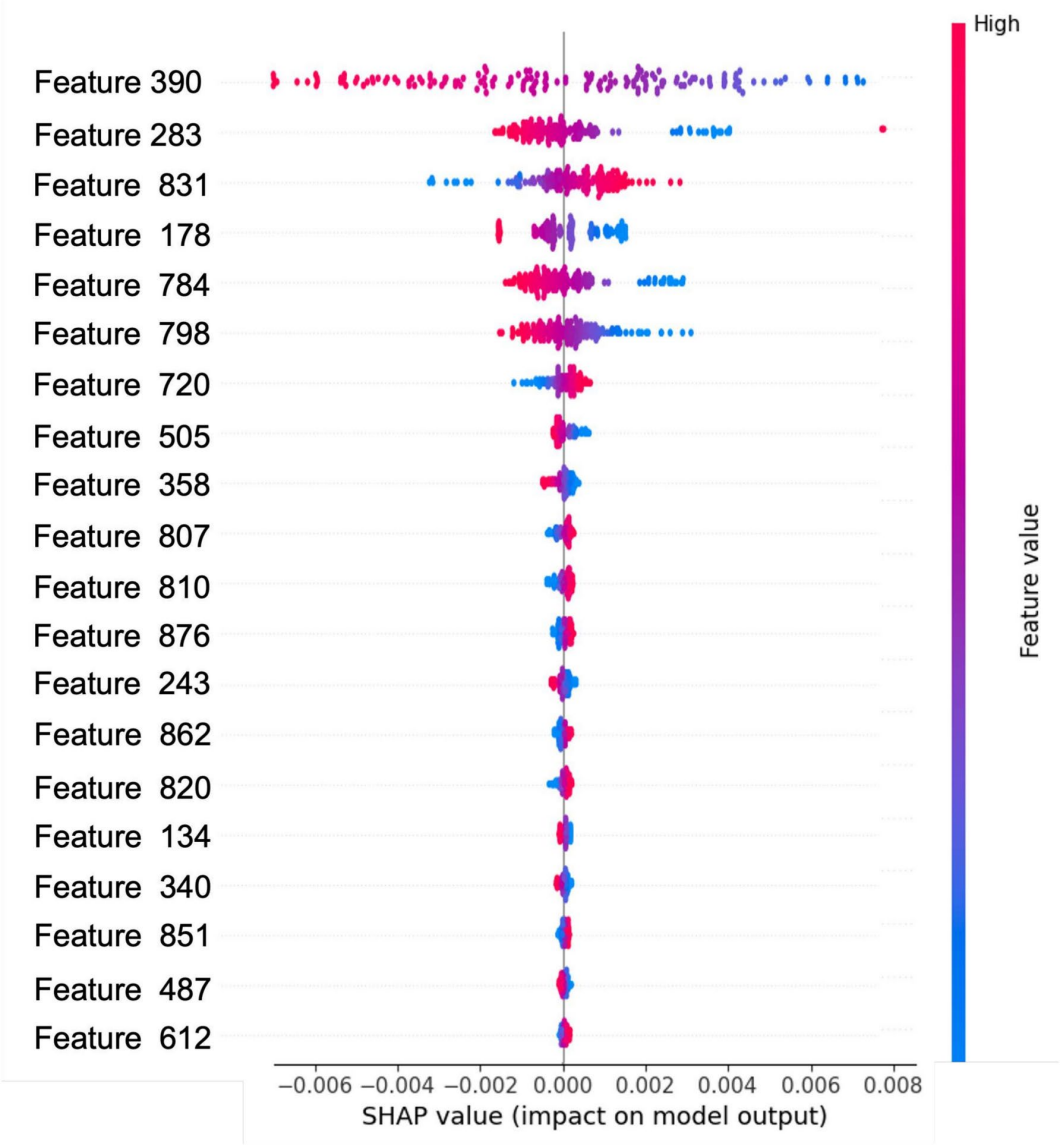
SHapley plot for regression branch (abrasion length).

27 $$\begin{aligned} \phi _j = \sum _{S \subseteq M \setminus \{j\}} \frac{|S|!(d - |S| - 1)!}{d!}(v(S \cup \{j\}) - v(S)) \end{aligned}$$

where $M = \{1, \ldots , d\}$ is the set of all features, $S$ is a subset of features not containing feature $j$, and $v(S)$ represents the contribution function, mapping a subset of features $S$ to a real number, which in this context, corresponds to the expected output of the model given only the features in $S$. $v(S)$ is often defined as:

28 $$\begin{aligned} v(S) = {\mathbb {E}}[f(x) \mid x_S = x_S^*] \end{aligned}$$

where $f(x)$ is the predictive model (in this case, the HHMLM, and $x_S$ represents the subset of input features. The Shapley value $\phi _j$ for each feature $j$ indicates the contribution of that feature to the model’s prediction. This allows for a detailed interpretation of the features driving the decision-making process. In Shapley plots, Red indicates that a feature positively influences the model’s prediction, increasing the likelihood of the chosen class. Blue indicates a feature has a negative influence, decreasing the likelihood of the chosen class or supporting an alternative class. The overall prediction $f(x^*)$ for a specific input $x^*$ can be decomposed using Shapley values as follows:

29 $$\begin{aligned} f(x^*) = \phi _0 + \sum _{j=1}^d \phi _j^* \end{aligned}$$

where $\phi _0 = {\mathbb {E}}[f({\varvec{x}})]$ is the average prediction across the dataset, and $\phi _j^*$ is the Shapley value for feature $j$ for the specific input $x^*$. The sum of the Shapley values for all features equals the difference between the specific prediction $f(x^*)$ and the average prediction $\phi _0$:

30 $$\begin{aligned} \sum _{j=1}^{d} \phi _j(v(S)) = f({x^*}) - {\mathbb {E}}[f({\varvec{x}})] \end{aligned}$$

The CNN or any machine learning model is considered a black box. Therefore, Shapley plots can help interpret the model’s classification/ regression tasks and increase the user’s confidence. The Shapley plot for damage type and location is illustrated in Fig. 11. For the damage type, frequency content features dominate the classification criteria, i.e., impact signals are picked up as low-frequency content indicated by red pixels in the lower portion of the SHAP values in Fig. 11-(a). Whereas for location, the peak wavelet coefficient and its position in the frequency-time domain contribute most to the damage localization as depicted in Fig. 11-(b). Further, the shapely plot for regression is in the form of feature contribution to the prediction as depicted in Fig. 12. The CNN part extracts these features from the input image. These plots give qualitative insights into model parameters that contribute to the predictions.

## Conclusions

- This study explores a practical approach to detecting and tracking damage in wind turbine blades using a unified hierarchical hybrid machine learning model (HHMLM) combined with acoustic emission (AE) data. Since AE generates large volumes of data due to its high sampling rate, the model is designed to work efficiently, making real-time monitoring more feasible even under noisy operational conditions.
- The model performed exceptionally well in distinguishing impact and PLB damage, achieving near-perfect classification accuracy (MCC of 0.99, F1-score of 1.0), while also localizing damage with high precision (MCC and F1 0.96). Even when tested with simulated operational noise, the model maintained its accuracy, proving that it can handle real-world conditions effectively.
- The HHMLM offers better accuracy and computational efficiency than traditional CNN models by integrating SVM. Also, the hierarchical structure of the model performs multiple tasks efficiently thereby reducing computational power requirements further.
- The HHMLM model results were analyzed using SHAP values, revealing a distinct separation between low and high-frequency features for damage classification. Damage localization was primarily influenced by factors such as the peak wavelet coefficient and its position in the frequency-time domain.

This research helps bridge the gap between laboratory studies and real-world applications by demonstrating a model that can work efficiently in practical settings. Future studies are planned on optimizing number of sensors, sensor placement and integrating wireless, TinyML-powered systems to make real-time monitoring of full-scale wind turbines even more accessible and reliable.

## Data Availability

All data produced or analyzed in this study are presented within the article.
